# Supplementary material for: Direct cell–cell contact between mature osteoblasts and osteoclasts dynamically controls their functions in vivo
Source: Nat Commun. 2018 Jan 19;9:300. doi: 10.1038/s41467-017-02541-w (PMC5775424; doi:10.1038/s41467-017-02541-w)
Supplement: Supplementary file 1 — Supplementary Information [file 41467_2017_2541_MOESM1_ESM.pdf]

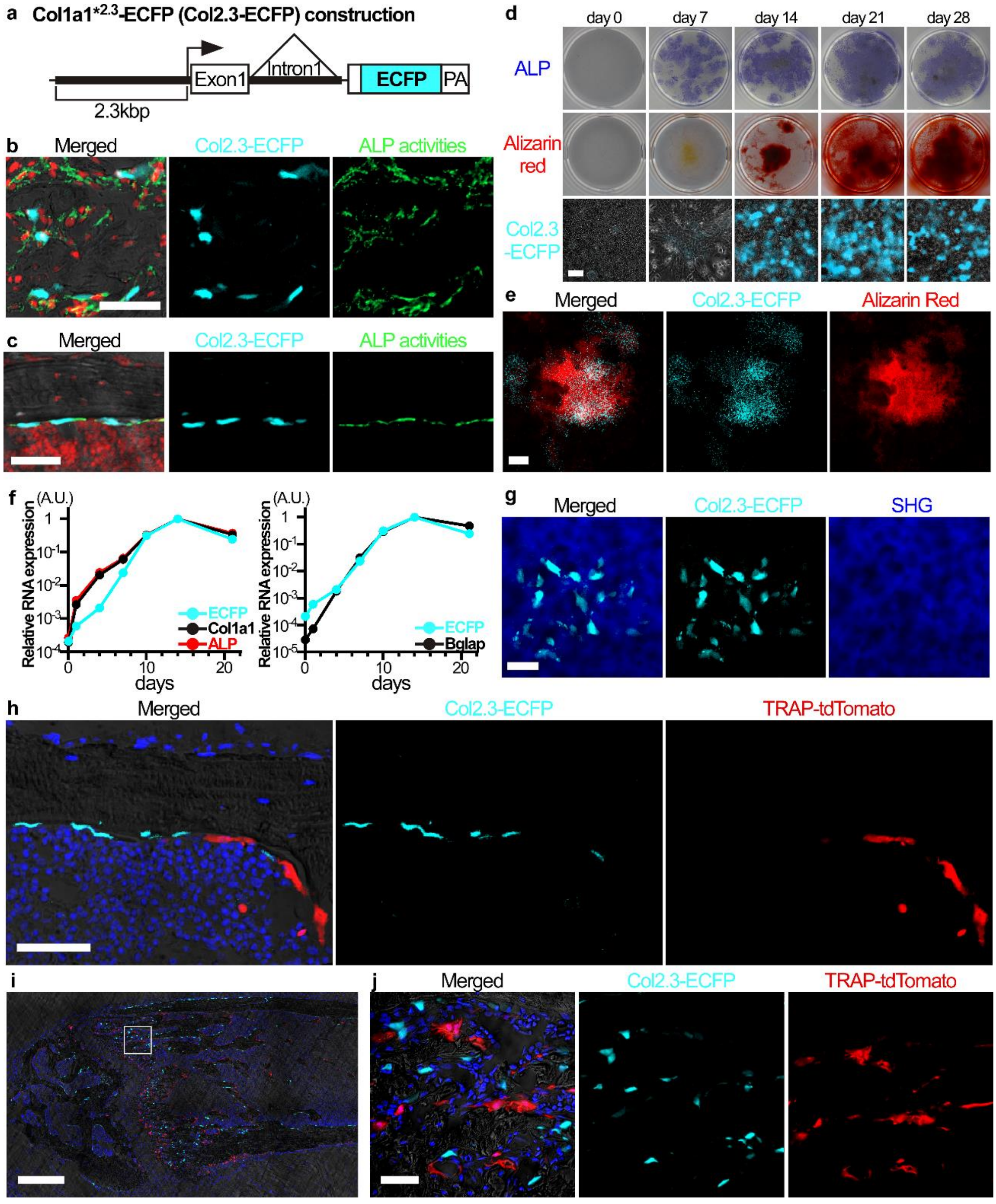

Supplementary Figure 1. Generation of fluorescent reporter mice expressing ECFP in the cytosol of mOBs.

(**a**) Construction of a mouse expressing the Col2.3-enhanced cyan fluorescent protein (ECFP) gene. (**b**) Histological analysis of the femoral, distal metaphyseal regions of Col2.3-ECFP transgenic mice. Col2.3-ECFP-positive cells (cyan) are visible along the bone surface. Immunohistochemical analysis of alkaline phosphatase (ALP) activity (green). Nuclei (7AAD, red). Scale bar, 50 µm. (**c**) Histological analysis of skull bone tissues of Col2.3-ECFP mice.

Immunohistochemical analysis of ALP activity (green). Nuclei (7AAD, red). Scale bar, 50 µm. (**d**) Osteoblastic differentiation culture of bone marrow stromal cells (BMSCs) derived from Col2.3-ECFP mice. ALP staining (top panels), Alizarin red staining (middle panels) and fluorescent images (bottom panels). Scale bar, 50 µm. (**e**) Representative day-14 images of an osteoblastic differentiation culture of BMSCs derived from Col2.3-ECFP mice. Mineralization (Alizarin red, red). Scale bar, 1,000 µm. (**f**) Relative expression levels of the genes encoding ECFP, Col1a1, ALP, and gamma-carboxyglutamic acid-containing protein (Bglap) in osteoblastic differentiation culture of BMSCs derived from Col2.3-ECFP mice, as derived by quantitative polymerase chain reaction (PCR) analysis. Data are presented as the mean values of three experiments; the expression levels are relative to those on day 14. (**g**) Representative, intravital, two-photon, microscopic MIP images of bone of Col2.3-ECFP mice held under control conditions. Cyan, mature osteoblasts (mOBs) expressing Col2.3-ECFP; blue, bone tissues (SHG). Scale bar, 50 µm. (**h**) Histological analysis of skull bone tissues of Col2.3-ECFP/TRAP-tdTomato mice. Col2.3-ECFP-positive osteoblasts (cyan) and TRAP-tdTomato-positive osteoclasts (red) are visible along the bone surface. Nuclei (DAPI, blue). Scale bar, 50 µm. (**i**) Histological analysis of a femoral metaphyseal region of Col2.3-ECFP/TRAP-tdTomato mice. Scale bar, 500 µm. (**j**) Magnified images of the boxed region shown in **i**. Scale bar, 50 µm.

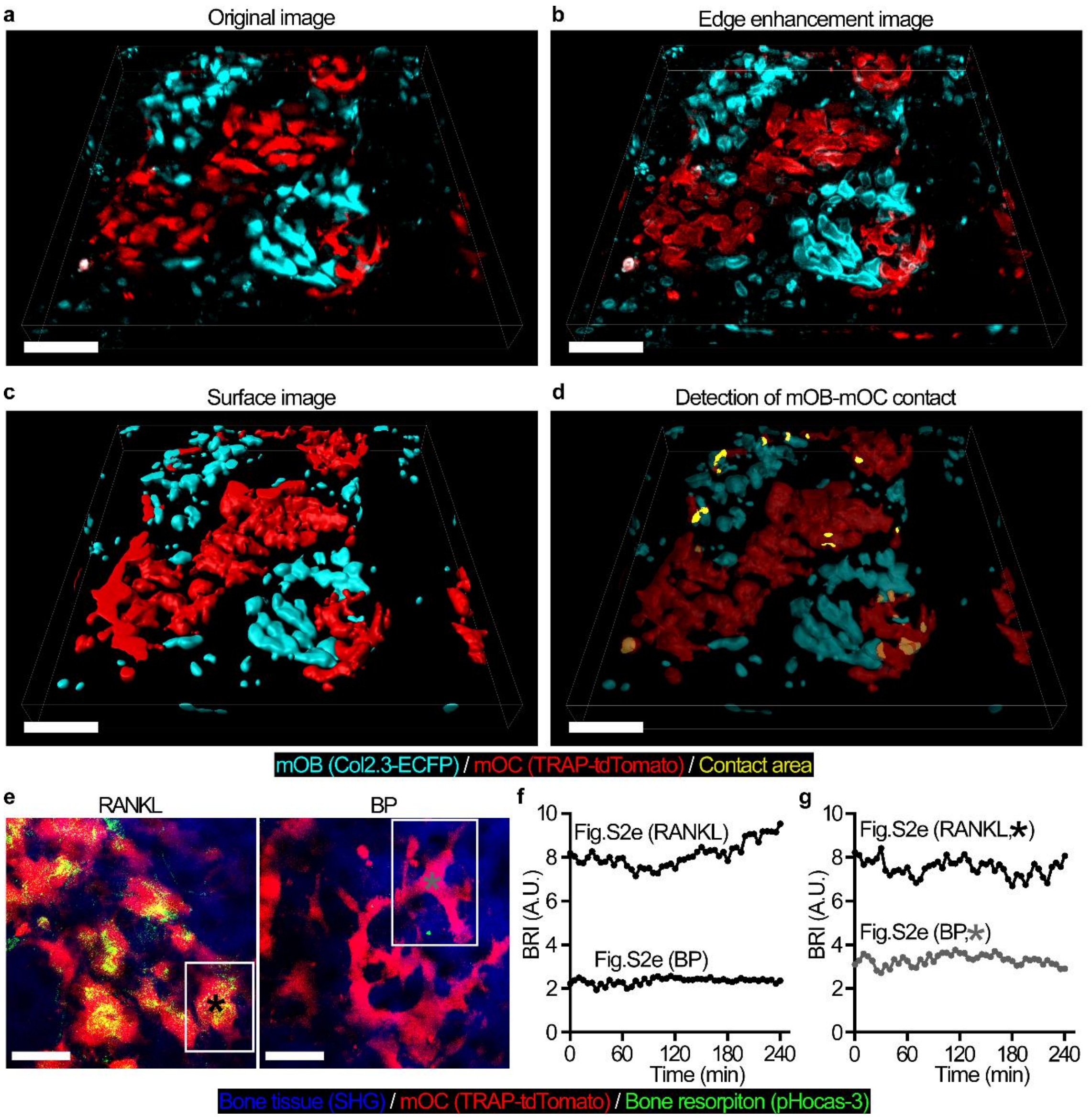

Supplementary Figure 2. Three-dimensional (3D) co-localization and BRI analyses. (**a**–**d**) The method used for 3D co-localization analysis. Scale bar, 100 µm. (**a**) A representative, intravital, two-photon microscopic 3D image of the skull bone tissue of a Col2.3-ECFP/TRAP-tdTomato mouse held under control conditions. Cyan, mOBs expressing Col2.3-ECFP; red, mOCs expressing TRAP-tdTomato. (**b**) A 3D edge-enhancement image of **a**. (**c**) 3D surface-rendering of mOBs and mOCs reconstructed from the edge enhancement Z-stack image of **b**. (**d**) The contact area was defined as the area of co-localization of mOBs and mOCs and is shown in yellow. (**e**) Representative MIP images of bone resorption activity in RANKL-treated and bisphosphonate-treated (BP-treated) TRAP-tdTomato mice, derived with the aid of the pH-sensing chemical probe pHocas-3. Green, fluorescent signals from pHocas-3; red, mOCs expressing TRAP-tdTomato; blue,

bone tissues (SHG). Scale bar, 50 µm. (**f**) The time courses of the BRIs of the visual fields shown in **e**. (**g**) The time courses of the BRIs for the mOCs identified with black and gray asterisks in the outlined region of **e**.

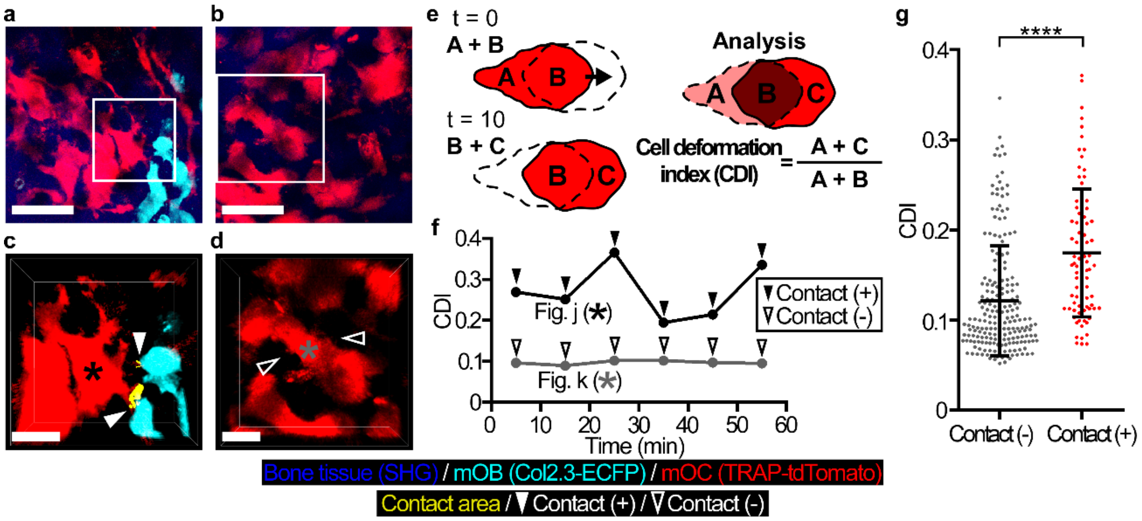

Supplementary Figure 3. Cell deformation index (CDI) analysis.

(**a**, **b**) Representative time-lapse MIP images of skull bone tissues from Col2.3-ECFP/TRAP-tdTomato mice under control conditions. Cyan, mOBs expressing Col2.3-ECFP; red, mOCs expressing TRAP-tdTomato; blue, bone tissues (SHG). Scale bar, 50 µm. (**a**) A representative image of a contact area. (**b**) A representative image of a colony area. (**c**, **d**) The 3D images yielded by co-localization analysis. Scale bar, 20 µm. (**c**; from the region outlined in **a**. **d**; from the region outlined in **b**.) The contact areas were those where mOBs and mOCs co-localized and are shown in yellow. The filled arrowheads show areas of mOB–mOC contact. The open arrowheads indicate separated mOBs and mOCs. (**e**) Cell deformation index (CDI) analysis. Cell shapes were semi-automatically extracted by the image analysis software, and three distinct areas were defined: occupied in the initial time frame (t = 0) (A), occupied in the final time frame (C), and overlapping between the two time frames (B). CDI was calculated as (A + C) / (A + B), representing the ratio of the areas changed during 10 min to the total cell area at t = 0. High or low CDI value correlates with the high or low motility of mOCs. (**f**) The time courses of the CDIs for the mOCs identified with black in **c** and gray asterisks in **d**. 3D co-localization analysis was also performed at 10-min intervals. (**g**) CDI of mOCs in contact, or not, with mOBs. Images were collected from 8 independent experiments. 3D co-localization analysis and CDI analysis were performed at 10-min intervals over 1h. Data points; n = 87 (mOCs in contact with mOBs), n = 207 (mOCs not in such contact). Data are presented as means ± SDs. ****$p < 0.0001$ (Mann–Whitney test).

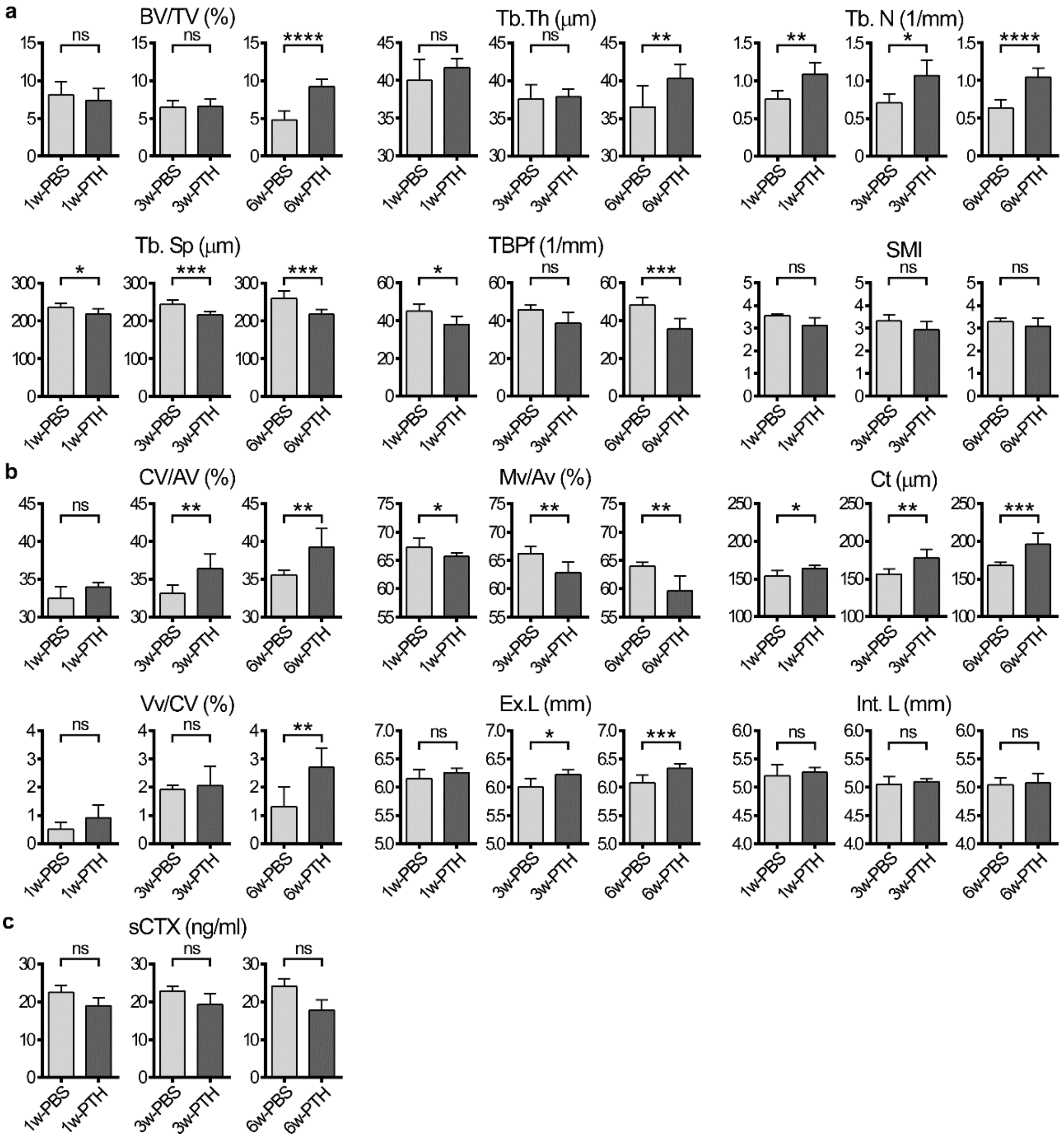

Supplementary Figure 4. Intermittent PTH treatment significantly increased both cortical and cancellous bone levels without elevating serum bone resorption markers *in vivo*.

(**a**, **b**) Micro-computed tomography (micro-CT) analysis of the distal, femoral metaphyseal regions of wild-type (WT) mice treated with PBS (control) or PTH (s.c., 40 µg/kg/day, 5 days/week). Treatment was initiated at 12 weeks of age, and the mice were analyzed 1, 3, and 6 weeks after treatment. (**a**) Analysis of trabecular bone structure. (**b**) Analysis of cortical bone structure. (**c**) The levels of a serum bone resorption marker (serum C-telopeptide of type 1 collagen, sCTX) in WT mice treated with PBS or PTH (s.c., 40 µg/kg/day, 5 days/week); n = 6–8 per group. Data are presented as means ± SDs. $^*p < 0.05$, $^{**}p < 0.01$, $^{***}p < 0.001$, $^{****}p < 0.0001$; ns, not significant (*t*-test or Mann–Whitney test).

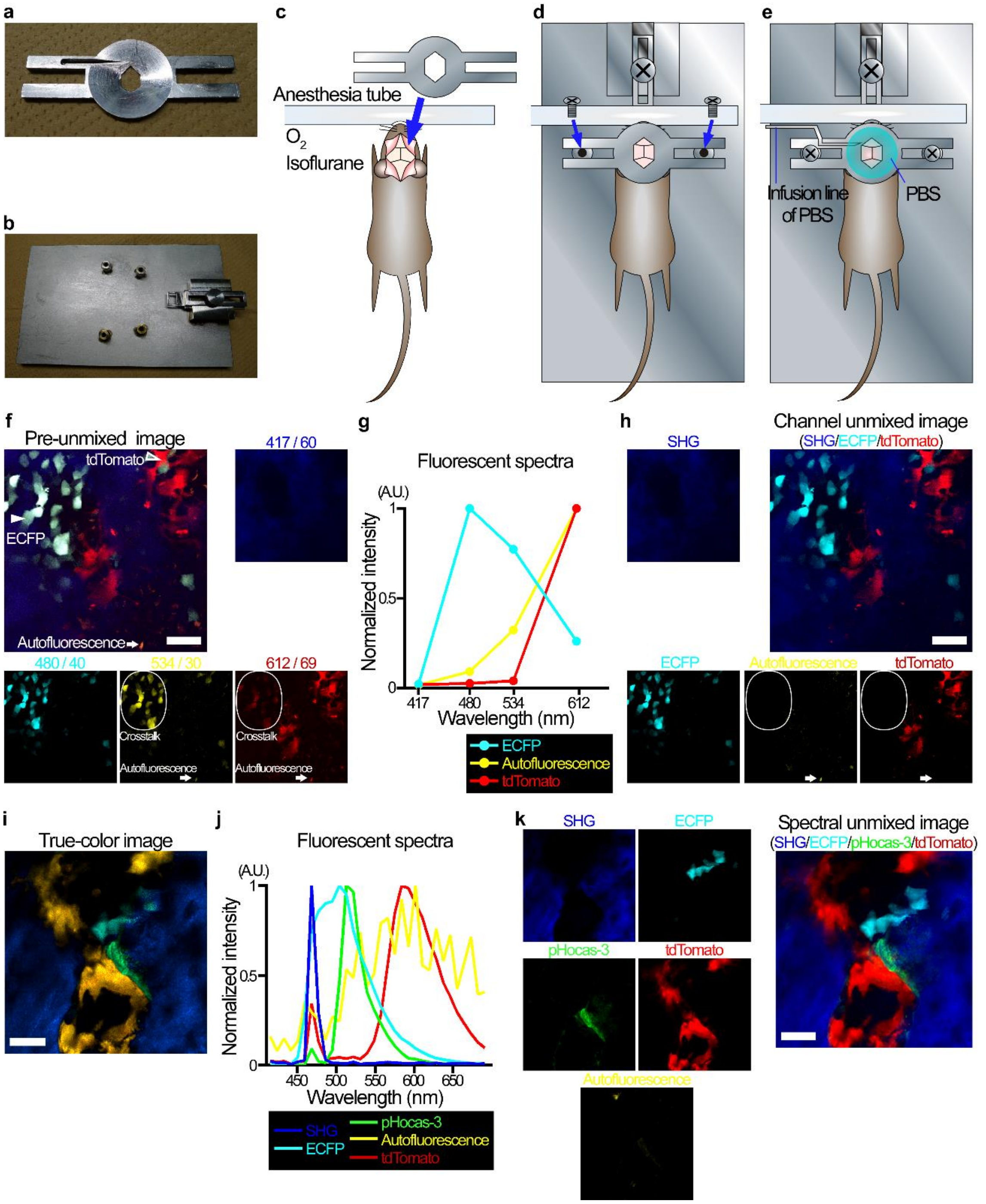

Supplementary Figure 5. The method used for intravital two-photon bone imaging. (**a**, **b**) An image of the stereotactic head holder (**a**) and the stage (**b**) used for intravital bone imaging. (**c**–**e**) A schematic showing how to fix the mouse skull using stereotactic instruments. (**c**) The frontoparietal region of the skull bone was exposed, and the stereotactic head holder was glued to the bone. (**d**) The head holder with the attached skull bone was fixed to the stereotactic stage using two screws. (**e**) A continuous infusion line was established in the

groove of the head holder, and the recess of the head holder was kept filled with PBS. The mouse thus prepared was placed in the original imaging box, and intravital bone imaging performed. (**f**) Representative MIP images of skull bone tissues from Col2.3-ECFP/TRAP-tdTomato mice were acquired using a two-photon microscope equipped with four external non-descanned (NDD) detectors; fluorescent images were acquired by each detector. The filled arrowheads, open arrowheads, arrows, and circles represent the ECFP signal, the tdTomato signal, autofluorescence, and crosstalk among the autofluorescence of mOBs and mOCs, respectively. Scale bar, 50 µm. (**g**) The cyan, red, and yellow lines represent the fluorescence spectra of ECFP, tdTomato, and autofluorescence, respectively. (**h**) The channel-unmixed fluorescence images shown in **f** and the spectral information shown in **g** were utilized to eliminate autofluorescence and fluorescence crosstalk. Scale bar, 50 µm. (**i**) A representative raw MIP image of bone-resorbing activity in skull bone tissue from a Col2.3-ECFP/TRAP-tdTomato mouse given pHocas-3 (a pH-sensing chemical probe); the image was acquired using a two-photon microscope equipped with specialized, internal multi-photomultiplier detectors. Scale bar, 25 µm. (**j**) The blue, cyan, green, red, and yellow lines represent the fluorescence spectra of SHG, ECFP, pHocas-3, tdTomato, and autofluorescence, respectively. (**k**) The spectral unmixed images shown in **i** and the spectral information shown in **j** were used to discriminate multi-fluorescent signals and to exclude autofluorescence. Scale bar, 25 µm.

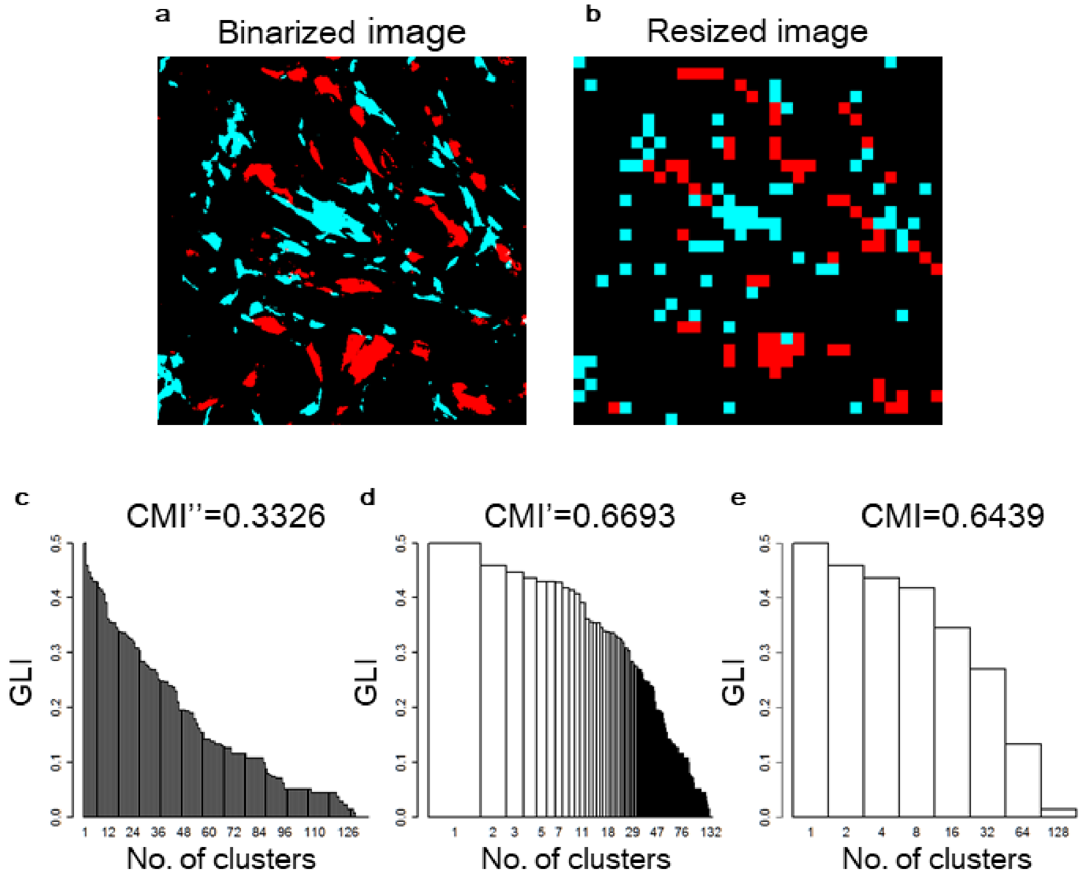

Supplementary Figure 6. Cell mixture index (CMI) and the approximation.

(**a**, **b**) A binarized and resized image of 6 weeks after PTH treatment. (**c**) A bar plot of the impurity values for every cluster set of image **b** without logarithmic weighting of the cluster numbers. (**d**) A bar plot of the impurity values for every cluster set of image **b** with logarithmic weighting of the cluster numbers. CMI value calculated by the equation (3) corresponds to the area of bars. (**e**) Approximation of calculating CMI.

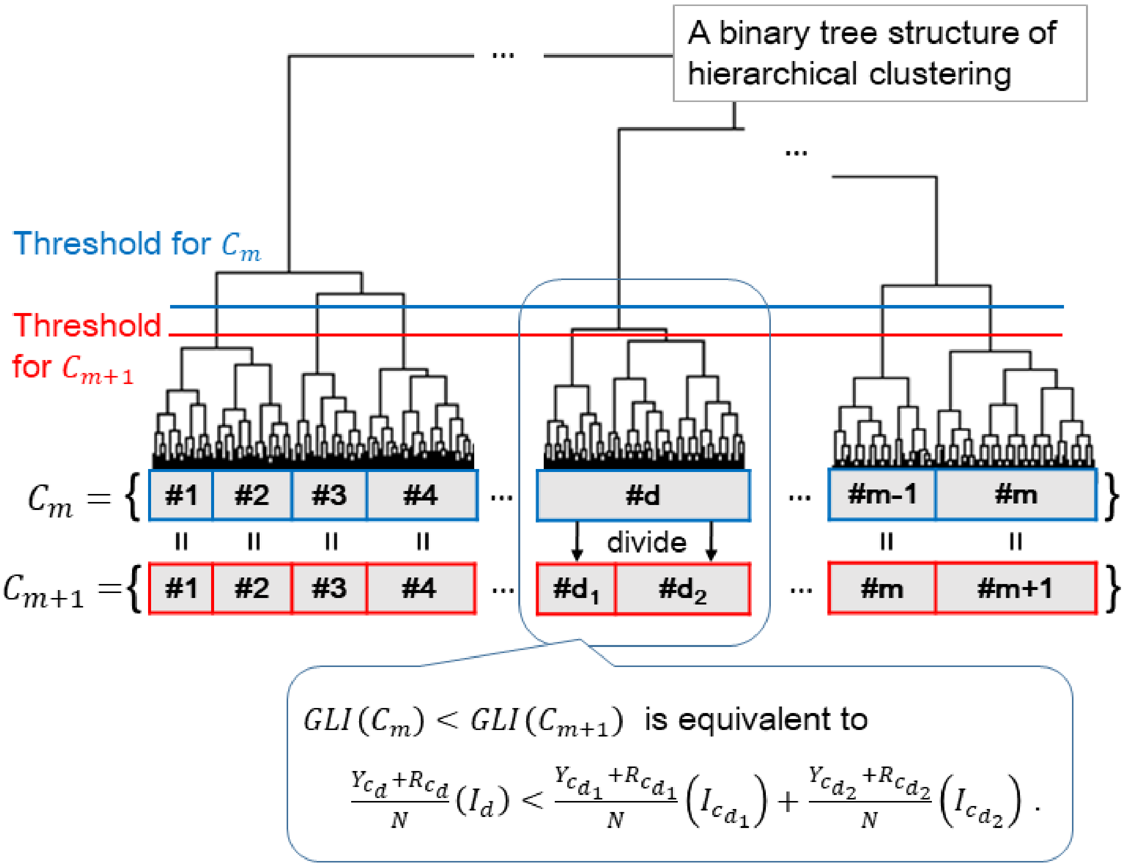

Supplementary Figure 7. Monotonically decreasing characteristics of Gini-like impurity with respect to the number of clusters.

To increase the number of clusters from m to m+1, a certain cluster is subdivided into two clusters.

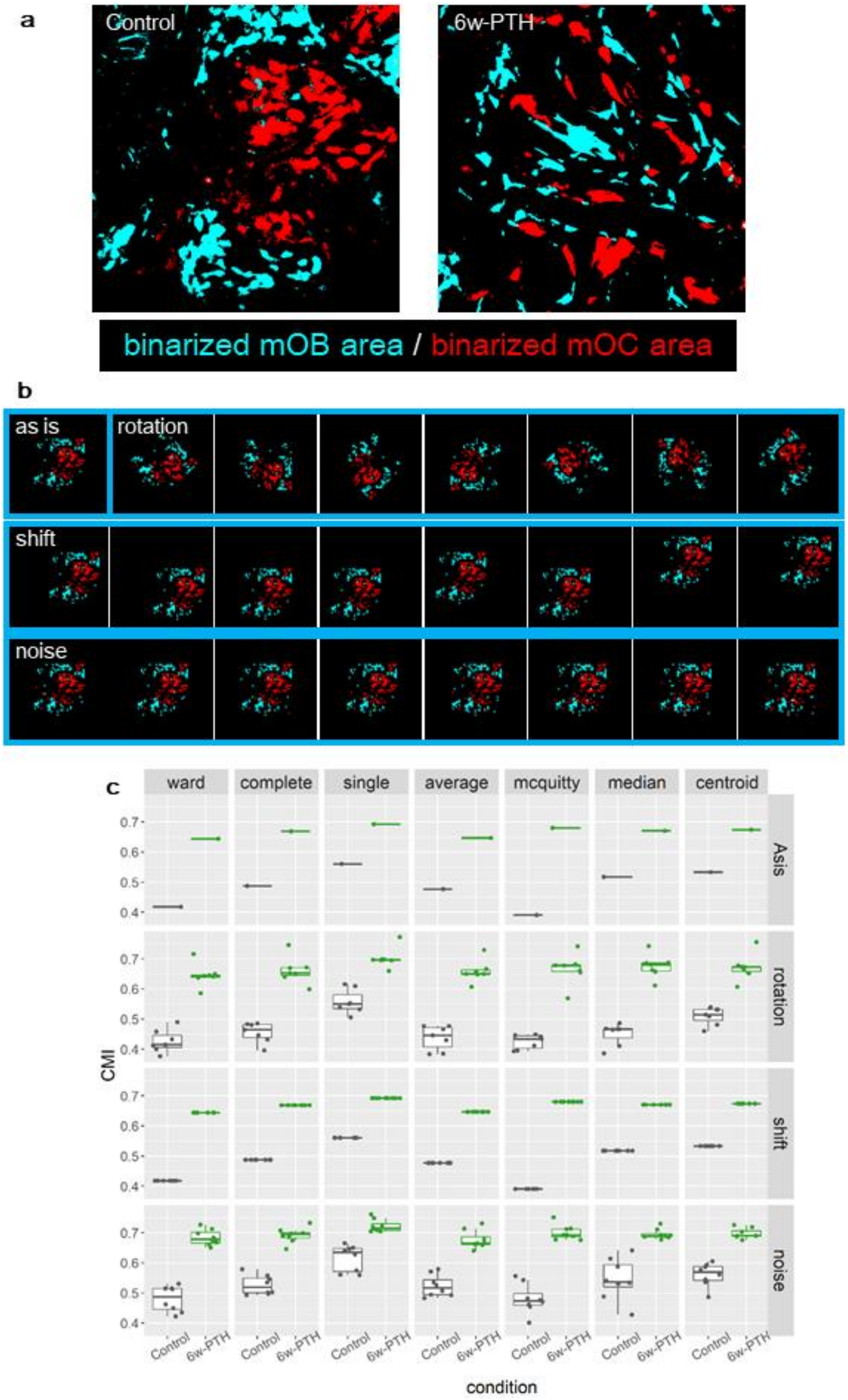

Supplementary Figure 8. Comparison of robustness of hierarchical clustering algorithms.

(**a**) Binarized image of control condition (left) and 6 weeks after PTH treatment (right). (**b**) Resized image of the control condition (as is) and examples of perturbed images (rotated, shifted, or after the addition of salt-and-pepper noise). (**c**) Comparison of CMI values using various hierarchical clustering algorithms against perturbations.

Supplementary Discussion

We discuss further about the formulation of cell mixture analysis used in this study.

First, we would like to explain the meanings of the equations (3) and (4) using the Supplementary Fig. 6. Supplementary Fig. 6a shows a binarized image (512x512 pixels) and Supplementary Fig. 6b shows a resized image (32x32 pixels) of Supplementary Fig. 6a. In Supplementary Fig. 6b, the total number of red and cyan pixels is 133, i.e., N=133. A bar plot of the impurity values for every cluster set is shown in Supplementary Fig. 6c (constructed without logarithmic weighting of the cluster numbers). In such a case, if we were to calculate a “CMI'' value in a manner similar to the way in which CMIs are usually calculated, the value would be the sum of the bars divided by 133 and multiplied by 2 (to normalize 0 to 1). This seems to underestimate the extent of mixture distribution. It is inappropriate to treat large and small changes equally; this is associated with a risk of miscalculation of cell areas. Therefore, we used the weighted averages (with respect to the cluster numbers) as shown in equation (3) and Supplementary Fig. 6d. Finally, we introduced an approximation [equation (4) and Supplementary Fig. 6e] to reduce computational costs, as described in the manuscript. In Supplementary Fig. 6e, the number of impurity calculations is drastically reduced, from N=133 to N'=8, without the loss of any quantification power (N' is the maximum number satisfying: $2^{N'} \leq \mathrm{N}$, i.e., $2^8 = 128 \leq 133$).

Second, the fact that the GLI decreases monotonically with an increasing number of clusters is proven below (via a contradiction approach). A prerequisite is the formation of a hierarchical clustering tree. Slicing of the tree using defined thresholds determines the cluster number. To increase the number of clusters from m to m+1, a certain cluster is subdivided into two clusters. Let d be the cluster that will be divided at step C_m and d_1 and d_2 the clusters thus obtained prior to step C_{m+1}. At this time, the other clusters are not at all affected, and neither the impurity nor weight of any cluster used to calculate the GLI would change in a cluster other than d (as shown in Supplementary Fig. 7):

Thus, if the GLI does not decrease monotonically, there exists a situation that satisfies $GLI(C_m) < GLI(C_{m+1})$. From equation (1):

$$GLI(C_m) = \sum_{i=1}^{m} \left(\frac{Y_{c_i} + R_{c_i}}{N} \left(I_{c_i} \right) \right) \qquad \text{(1, reshown)}$$

considering that $GLI(C_m) < GLI(C_{m+1})$ is equivalent to a comparison between the weighted impurity of cluster d at C_m and those of d_1 and d_2 at $C_m + 1$; that is,

$$\frac{Y_{c_d}+R_{c_d}}{N}\left(I_d\right) < \frac{Y_{c_{d_1}}+R_{c_{d_1}}}{N}\left(I_{c_{d_1}}\right) + \frac{Y_{c_{d_2}}+R_{c_{d_2}}}{N}\left(I_{c_{d_2}}\right) \qquad \text{(i).}$$

Now, cluster d_1 and cluster d_2 are subsets of cluster d, and the following equations (ii, iii) are always true:

$$Y_{c_d} = Y_{c_{d_1}} + Y_{c_{d_2}} \qquad \text{(ii)}$$

$$R_{c_d} = R_{c_{d_1}} + R_{c_{d_2}} \qquad \text{(iii).}$$

Using

$$I_{c_i} = 1 - \left(\frac{Y_{c_i}^2+R_{c_i}^2}{\left(Y_{c_i}+R_{c_i}\right)^2}\right) \qquad \text{(2, reshown),}$$

as the definition of impurity, we can transform inequality (i) into inequality (iv) in the following way:

$$\frac{Y_{c_d}+R_{c_d}}{N}\left(\frac{2Y_{c_d}R_{c_d}}{\left(Y_{c_d}+R_{c_d}\right)^2}\right) < \frac{Y_{c_{d_1}}+R_{c_{d_1}}}{N}\left(\frac{2Y_{c_{d_1}}R_{c_{d_1}}}{\left(Y_{c_{d_1}}+R_{c_{d_1}}\right)^2}\right) + \frac{Y_{c_{d_2}}+R_{c_{d_2}}}{N}\left(\frac{2Y_{c_{d_2}}R_{c_{d_2}}}{\left(Y_{c_{d_2}}+R_{c_{d_2}}\right)^2}\right) \qquad \text{(iv).}$$

Now, multiplied by N and move the terms on the right to the left as follows:

$$\frac{2Y_{c_d}R_{c_d}}{\left(Y_{c_d}+R_{c_d}\right)} - \frac{2Y_{c_{d_1}}R_{c_{d_1}}}{Y_{c_{d_1}}+R_{c_{d_1}}} - \frac{2Y_{c_{d_2}}R_{c_{d_2}}}{Y_{c_{d_2}}+R_{c_{d_2}}} < 0. \qquad \text{(v).}$$

Substitute equations (ii) and (iii) into inequality (v) as follows:

$$\frac{2Y_{c_d}R_{c_d}}{\left(Y_{c_d}+R_{c_d}\right)} - \frac{2Y_{c_{d_1}}R_{c_{d_1}}}{Y_{c_{d_1}}+R_{c_{d_1}}} - \frac{\left(Y_{c_d}-Y_{c_{d_1}}\right)\left(R_{c_d}-R_{c_{d_1}}\right)}{\left(Y_{c_d}+R_{c_d}\right)-\left(Y_{c_{d_1}}+R_{c_{d_1}}\right)} < 0. \qquad \text{(vi).}$$

After ensuring that all fractions have common denominators, we obtain the following inequality:

$$\left(Y_{c_d}+R_{c_d}\right)\left(Y_{c_{d_1}}+R_{c_{d_1}}\right)\left(\left(Y_{c_d}+R_{c_d}\right)-\left(Y_{c_{d_1}}+R_{c_{d_1}}\right)\right)\left(Y_{c_d}R_{c_{d_1}}-Y_{c_{d_1}}R_{c_d}\right)^2 < 0 \qquad \text{(vii).}$$

In inequality (vii), the 1st, 2nd, and 4th terms are obviously positive. To satisfy the inequality, the 3rd term must thus be negative. However, because cluster d_1 and cluster # are subsets of cluster d, $Y_{c_d} > Y_{c_{d_1}}$ and $R_{c_d} > R_{c_{d_1}}$ are always true. Thus, the 3rd term is always positive, and inequality (vii) has no solution. It follows that the GLI monotonically decreases by the number of clusters.

Finally, we mention the reason why we chose the hierarchical clustering with Ward's method to analyze the degree of mOB–mOC mixture distribution. Clustering methods search for data patterns by joining or dividing elements. Various clustering methods have been proposed, and all have both advantages and disadvantages. We used hierarchical clustering because this is the simplest method, as relatively few parameters must be defined in advance. In addition, availability of monotonically decreasing characteristics of GLI is also one of the main reasons for the choice. Then, in order to evaluate the robustness of several hierarchical

clustering methods, we present some patterns exhibiting small perturbations of the original images. We evaluated hierarchical clustering algorithms (Ward’s method, complete linkage, single linkage, average linkage, McQuitty’s method, median linkage, and centroid linkage) to explore differences in the CMIs generated. Supplementary Fig. 8a shows the typical patterns of control and PTH 6-week images, respectively. Supplementary Fig. 8b contains examples of perturbed images (rotated, shifted, or after the addition of salt-and-pepper noise). Supplementary Fig. 8c shows the CMI values, calculated using various algorithms, of the perturbed images. Ward’s method was the most robust [the leftmost column of Supplementary Fig. 8c] (McQuitty’s method shows also good performance). Ward’s method is widely used in the field of data mining. The computation time is slightly longer than that required for single linkage or complete linkage, but it generates well-balanced clusters even when outliers are present.
