# Supplementary material for: Direct cell–cell contact between mature osteoblasts and osteoclasts dynamically controls their functions in vivo
Source: Nat Commun. 2018 Jan 19;9:300. doi: 10.1038/s41467-017-02541-w (PMC5775424; doi:10.1038/s41467-017-02541-w)
Supplement: Supplementary file 3 — Description of Additional Supplementary Files [file 41467_2017_2541_MOESM3_ESM.docx]

**Description of Additional Supplementary Files**

File Name: Supplementary Movie 1

Description: Intravital two-photon imaging of skull bone tissues from Col2.3- ECFP/TRAP-tdTomato mice held under control conditions. Sequential images of the same visual field are shown. Cyan, mature osteoblasts (mOBs) expressing Col2.3-ECFP; red, mature osteoclasts (mOCs) expressing TRAP-tdTomato; blue, bone tissues (SHG). The arrowheads indicate mOCs with synapse-like projections toward mOBs. Scale bar, 100 µm. Playback speed = 4500×.

File Name: Supplementary Movie 2

Description: Intravital two-photon imaging of bone-resorbing activity in skull bone tissues from Col2.3-ECFP/TRAP-tdTomato mice held under control conditions. Mice were treated with pHocas-3, a pH-sensing chemical probe. Sequential images of the same visual field were acquired under control conditions. Green, fluorescent signals from pHocas-3; Cyan, mOBs expressing Col2.3-ECFP; red, mOCs expressing TRAPtdTomato; blue, bone tissues (SHG). Scale bar, 25 µm. Playback speed = 4500×.

File Name: Supplementary Movie 3

Description: Intravital two-photon imaging of skull bone tissues from Col2.3- ECFP/TRAP-tdTomato mice held under control conditions. Sequential images of the same visual field were acquired, followed by 3D co-localization analysis. Cyan, mOBs expressing Col2.3-ECFP; red, mOCs expressing TRAP-tdTomato; blue, bone tissues (SHG); yellow, the areas of co-localization of mOBs and mOCs. Scale bar, 100 µm. Playback speed = 4500×

File Name: Supplementary Movie 4

Description: Intravital two-photon imaging of skull bone tissues from Col2.3- ECFP/TRAP-tdTomato mice treated with PTH for 1 week. Mice were injected s.c. with parathyroid hormone (PTH, 40 µg/kg/day, daily for 5 days over 1 week). Sequential images of the same visual field were acquired, and 3D co-localization analysis was then performed. Cyan, mOBs expressing Col2.3-ECFP; red, mOCs expressing TRAP-tdTomato; blue, bone tissues (SHG); yellow, the areas of co-localization of mOBs and mOCs. Scale bar, 100 µm. Playback speed = 4500×.

File Name: Supplementary Movie 5

Description: Intravital two-photon imaging of skull bone tissues from Col2.3- ECFP/TRAP-tdTomato mice treated with PTH for 3 weeks. Mice were given PTH (40 µg/kg/day, 5 days/week) via s.c. injection for 3 weeks. Sequential images of the same visual field were acquired, and 3D co-localization analysis was then performed. Cyan, mOBs expressing Col2.3-ECFP; red, mOCs expressing TRAP-tdTomato; blue, bone tissues (SHG); yellow, the areas of co-localization of mOBs and mOCs. Scale bar, 100 µm. Playback speed = 4500×.

File Name: Supplementary Movie 6

Description: Intravital two-photon imaging of skull bone tissues from Col2.3- ECFP/TRAP-tdTomato mice treated with PTH for 6 weeks. Mice were given PTH (40 µg/kg/day, 5 days/week) via s.c. injection for 6 weeks. Sequential images of the same visual field were acquired, and 3D co-localization analysis was then performed. Cyan, mOBs expressing Col2.3-ECFP; red, mOCs expressing TRAP-tdTomato; blue, bone tissues (SHG); yellow, the areas of co-localization of mOBs and mOCs. Scale bar, 100 µm. Playback speed = 4500×.

File Name: Supplementary Movie 7

Description: Intravital two-photon imaging of bone-resorbing activity in skull bone tissues from Col2.3-ECFP/TRAP-tdTomato mice held under control conditions. Mice were given pHocas-3 via s.c. injection over 3 days. Green, fluorescent 3 signals from pHocas-3; Cyan, mOBs expressing Col2.3-ECFP; red, mOCs expressing TRAPtdTomato; blue, bone tissues (SHG). Scale bar, 50 µm. Playback speed = 4500×.

File Name: Supplementary Movie 8

Description: Intravital two-photon imaging of bone-resorbing activity in skull bone tissues from Col2.3-ECFP/TRAP-tdTomato mice treated with PTH for 1 week. Mice were injected s.c. with PTH (40 µg/kg/day, daily for 5 days over 1 week) and pHocas-3 (daily for 3 days). Green, fluorescent signals from pHocas-3; Cyan, mOBs expressing Col2.3- ECFP; red, mOCs expressing TRAP-tdTomato; blue, bone tissues (SHG). Scale bar, 50 µm. Playback speed = 4500×.

File Name: Supplementary Movie 9

Description: Intravital two-photon imaging of bone-resorbing activity in skull bone tissues from Col2.3-ECFP/TRAP-tdTomato mice treated with PTH for 3 weeks. Mice were given PTH (40 µg/kg/day, 5 days/week) via s.c. injection for 3 weeks and pHocas-3 via s.c. injections on each of 3 days. Green, fluorescent signals from pHocas-3; Cyan, mOBs expressing Col2.3-ECFP; red, mOCs expressing TRAP-tdTomato; blue, bone tissues (SHG). Scale bar, 50 µm. Playback speed = 4500×.

File Name: Supplementary Movie 10

Description: Intravital two-photon imaging of bone-resorbing activity in skull bone tissues from Col2.3-ECFP/TRAP-tdTomato mice treated with PTH for 6 weeks. Mice were given PTH (40 µg/kg/day, 5 days/week) via s.c. injection for 6 weeks and pHocas-3 via s.c. injection over 3 days. Green, fluorescent signals from pHocas-3; Cyan, mOBs expressing Col2.3-ECFP; red, mOCs expressing TRAP-tdTomato; blue, bone tissues (SHG). Scale bar, 50 µm. Playback speed = 4500×.
